# Effectiveness and Safety of Oral Chinese Patent Medicines Combined with Chemotherapy for Gastric Cancer: A Bayesian Network Meta-Analysis

**DOI:** 10.1155/2020/8016531

**Published:** 2020-08-26

**Authors:** Xiaona Lu, Yawei Zheng, Fang Wen, Wenjie Huang, Peng Shu

**Affiliations:** ^1^First School of Clinical Medicine, Nanjing University of Chinese Medicine, Nanjing 210029, China; ^2^Oncology Department, Jiangsu Province Hospital of Chinese Medicine, Nanjing 210029, China

## Abstract

**Objectives:**

This network meta-analysis (NMA) was designed to assess the comparative effectiveness and safety of oral Chinese patent medicines combined with chemotherapy for gastric cancer on the National Basic Medical Insurance Drugs List of China.

**Methods:**

A comprehensive literature search was performed in seven electronic databases from their inception to February 25, 2020, aiming to collect all related randomized controlled trials (RCTs) to evaluate the effectiveness and safety of oral Chinese patent medicines as an adjuvant for gastric cancer. Two researchers independently screened the literature, extracted data, and assessed the risk of bias of included studies using the Cochrane Risk of Bias Scale. NMA was then performed by using STATA 16.0 software and ADDIS 1.16.8 software.

**Results:**

Finally, 30 RCTs were included, involving seven kinds of oral Chinese patent medicines, with a total of 2602 patients. For improvement of clinical efficacy, Bazhen granule combined with chemotherapy was ranked first for effectiveness, followed by the Cinobufacin capsule combined with chemotherapy and Xiao'aiping tablet combined with chemotherapy. Meanwhile, Bazhen granules combined with chemotherapy also were ranked first in reducing gastrointestinal reactions. In terms of improving performance status, the Xiao'aiping tablet was the best and significantly better than other oral Chinese patent medicines. Besides, the Zhenqi Fuzheng granule combined with chemotherapy was best for reducing the incidence of leucopenia.

**Conclusions:**

Since only one RCT of Bazhen granule was included in this study for analysis, its statistical efficiency is low. Therefore, this study recommends that the Cinobufacin capsule combined with chemotherapy should be a priority in improving clinical efficacy. In terms of improving patients' quality of life, Xiao'aiping tablet is the best choice. Safety was best for Zhenqi Fuzheng granule and Bazhen granule combined with chemotherapy. Limited by the quantity, quality, and possible bias of included studies, the above conclusions need to be further verified by more high-quality RCTs.

## 1. Introduction

Gastric cancer is one of the leading causes of cancer-related death worldwide, and its incidence is sixth in the world's cancer. China is a country with a high incidence of gastric cancer, and China accounts for nearly half the world's gastric cancer burden [1]. According to the statistics of China National Cancer Center, gastric cancer ranks among the top three in terms of morbidity and mortality and is a malignant tumour with serious harm [2]. At present, surgery is considered to be the only radical treatment. However, gastric cancer has the characteristics of high incidence, a high metastatic rate, high mortality, low early diagnosis rate, low radical resection rate, and low five-year survival rate. Therefore, chemotherapy plays a vital role in prolonging the survival time of gastric cancer patients [3]. Although chemotherapy can extend the survival period of patients, its adverse reactions also seriously affect the quality of life of patients and are even challenging to tolerate chemotherapy. Traditional Chinese medicine (TCM) has been used to treat cancer for thousands of years. As an essential part of complementary and alternative therapy, it has become one of the vital means of comprehensive treatment of gastric cancer. In recent years, researches on the treatment of gastric cancer with TCM have shown that it has the effects of improving clinical efficacy, resisting recurrence and metastasis, improving quality of life, reducing toxic and side effects of radiotherapy, and chemotherapy and enhancing immunity [4]. Therefore, TCM adjuvant therapy is of considerable significance in improving the constitution of gastric cancer patients, improving the completion rate and efficiency of chemotherapy, etc. Besides, compared with Chinese herbal pieces, oral Chinese patent medicine has the advantages of convenient administration and accurate dosage, which have been widely used in clinical trials. Oral Chinese patent medicine can often play a better role in the treatment of gastric cancer, which can be used in combination with chemotherapy for patients in generally good condition. In contrast, for patients without chemotherapy indications, it can be used alone to achieve the purpose of disease control. Also, some adjuvant oral Chinese patent medicines can be used to relieve various discomfort symptoms of gastric cancer patients, such as pain, belching, acid reflux, hematochezia, and emaciation and can reduce the adverse reactions after chemotherapy and improve the immunity of patients to improve the quality of life of gastric cancer patients [5].

As an extension of traditional meta-analysis, network meta-analysis has the advantage of combining multiple processing and indirect comparison evidence, ranking the sufficient probability of interventions, and providing more comprehensive and valuable information for clinical decision-making [6]. Although many trials have compared the efficacy and safety of oral Chinese patent medicine in the treatment of gastric cancer, there is a lack of head-to-head comparisons between different oral Chinese patent medicines, and its relative advantages have not been well understood. Therefore, to confirm the best therapy, this study uses NMA to compare the efficacy and safety of multiple oral Chinese patent medicines combined with chemotherapy in the treatment of gastric cancer, aiming to provide evidence-based medicine basis for clinical decision-making.

## 2. Materials and Methods

This NMA was conducted by the PRISMA NMA Statement [7]. A completed PRISMA 2015 network meta-analysis checklist was included as supplementary material (Table S1).

### 2.1. Eligibility Criteria

#### 2.1.1. Types of Studies

Studies included are randomized controlled trials (RCTs), regardless of blinding. The languages are limited to Chinese and English.

#### 2.1.2. Types of Participants

Patients with definite pathological diagnosis of gastric cancer have unlimited stages, age ≥ 18 years old, no limitation of gender, race, nationality, etc. There is at least one measurable clinical or imaging observation index, with Karnofsky (KPS) score ≥ 60 or Eastern Cooperative Oncology Group (ECOG) score of 0–2. There is no chemotherapy contraindication before treatment, and there is no obvious abnormality in liver and kidney function, haematology, and electrocardiograph (ECG).

#### 2.1.3. Types of Interventions

Interventions involving oral Chinese patent medicines combined with chemotherapy for the treatment of gastric cancer are eligible. The control groups include chemotherapy alone or another oral Chinese patent medicine combined with chemotherapy. These oral Chinese patent medicines are recommended by the *Clinical Practice Guidelines of Chinese Medicine in Oncology* and included in the National Basic Medical Insurance Drugs List of China, specifically Bailing capsule, Jianpi Yishen granule, Zhenqi Fuzheng capsule/granule/tablet, Bazhen granule/capsule/pill/tablet, Buzhong Yiqi pill, Shiquan Dabu pill, Xiao'aiping tablet/capsule/drop pill, Cinobufacin tablet/capsule, Antike capsule, Pingxiao capsule/tablet, Andolin capsule, Yangyin Shengxue mixture, Shiyiwei Shengqi tablet/capsule, Shenqi Shiyiwei granule, and Kanglaite soft capsule [8].

#### 2.1.4. Types of Outcome Measures

The primary effectiveness outcome was the objective response rate (ORR). ORR was evaluated according to Response Evaluation Criteria in Solid Tumors (RECIST 1.1) [9]. ORR = [Complete Response (CR) + Partial Response (PR) ]/total cases × 100%. The secondary outcome was performance status. Performance status was assessed by the Karnofsky (KPS) score. After treatment, the KPS score increased by more than 10 points was considered effective. The safety outcome was adverse drug reactions (ADRs) involving the incidence of leucopenia and gastrointestinal reaction. The incidence of ADRs = number of ADRs/total cases × 100%.

### 2.2. Exclusion Criteria

(1) It was associated with any other primary tumours, such as lung cancer; (2) it was combined with other interventions, such as acupuncture and other traditional Chinese medicine treatments; (3) it was repeatedly published literature; (4) no valid data were reported for analysis or the data were not credible.

### 2.3. Information Sources and Search

We searched in seven electronic databases including the Cochrane Library, PubMed, Embase, China National Knowledge Infrastructure (CNKI), WanFang Data, China Science and Technology Journal Database (CSTJ), and China Biology Medicine disc (CBMdisc) from their inception to February 25, 2020, to collect RCTs of oral Chinese patent medicine combined with chemotherapy in the treatment of gastric cancer. The search is carried out by the combination of subject words and free words. Search terms include “stomach neoplasms,” “gastric cancer,” “stomach cancer,” “medicine” (name of each oral Chinese patent medicine), and “randomized controlled trial”. Taking PubMed as an example, its specific search strategy is shown in Table S2.

### 2.4. Study Selection and Data Extraction

Endnote X9 software was used to manage literature and delete duplicate literature. Then, two researchers independently screened literature, extracted data, and cross-checked them according to the eligibility criteria. After removing the apparently unrelated studies by reading the title, further, they read the abstract and the full text to determine whether to include the remaining studies. If there are differences in the implementation process, they shall be solved through discussion or consultation with the tutor.

The data of included studies were extracted into the designed Microsoft Excel sheet, containing the first author, publication year, sample size, baseline characteristics (TNM stage, sex, age), intervention measures, course of treatment, and outcomes.

### 2.5. Risk of Bias within Individual Studies

Two researchers assessed the risk of bias within individual studies independently by using the Cochrane Risk of Bias (Scale) [10]. The items mainly include the following seven aspects: (1) random sequence generation (selection bias), (2) allocation concealment (selection bias), (3) blinding of participants and personnel (performance bias), (4) blinding of outcome assessment (detection bias), (5) incomplete outcome data (attrition bias), (6) selective reporting (reporting bias), and (7) other biases. The evaluation level of bias risk is split into “low risk,” “unclear risk,” and “high risk.” If there gets some inconsistency, it shall be solved through collective discussion or consultation with the tutor.

### 2.6. Statistical Analysis

Review Manager 5.3 software, STATA 16.0 software, and Aggregate Data Drug Information System (ADDIS) 1.16.8 software were used for statistical analysis. In this study, outcome data types are all dichotomous variables, so the odds ratio (OR) and its 95% confidence interval (CI) were used as the effect. Review Manager 5.3 was used for literature quality evaluation. Stata 16.0 software was used to analyse the heterogeneity and draw the evidence network graph of each outcome. In the network graph, the size of treatment nodes reflects the number of patients randomly allocated to each treatment, and the thickness of edges represents the number of studies underlying each comparison; A comparison-adjusted funnel plot was made to evaluate whether there was a publication bias in the study.

The heterogeneity between the results of each direct comparison was analysed by the chi-square test (the test level was *α* = 0.1). And the size of heterogeneity was quantitatively determined by combining with *I*^*2*^. *I*^*2*^ ≤ 50%, indicating that the heterogeneity between the research results is small, and the fixed-effect model was used for meta-analysis; otherwise, the heterogeneity is considerable; on the premise of excluding clinical heterogeneity, the random effect model can be used for meta-analysis [11]. Apparent clinical heterogeneity was treated by subgroup analysis or sensitivity analysis or only descriptive analysis. When there is a closed-loop structure between the interventions, it is necessary to perform the inconsistency test. Judging by the inconsistency factor (IF), when the IF value 95% CI contains 0, it means that the direct evidence is consistent with the indirect evidence.

ADDIS 1.16.8 software which is based on the Bayesian framework using the Markov chain Monte Carlo (MCMC) method for prior assessment and implementation was used for the network meta-analysis [12]. Four Markov chains are used to set the initial value. The variance scaling factor of the model is 2.5, the thinning interval is 10, the tuning iterations are 20000, and the simulation iterations are 50000. When the potential scale reduced factor (PSRF) tends to 1, the convergence degree is satisfied. Finally, the results of network meta-analysis are presented in tabular form, and the probability of each intervention becoming the best one is offered by ranking probability.

## 3. Results

### 3.1. Study Selection and Study Characteristics

**Figure 1:**
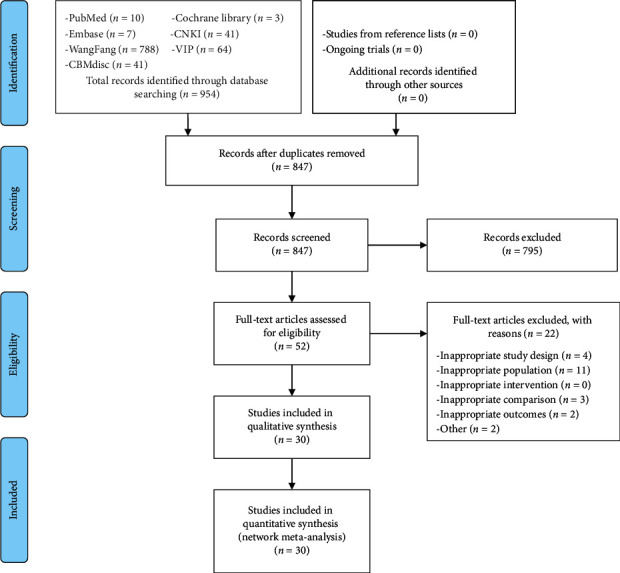
The PRISMA flow diagram of study selection (*n*: number of articles; CNKI: the China National Knowledge Infrastructure Database; WanFang: the WanFang Database; CSTJ: the China Science and Technology Journal Database; CBMdisc: the China Biology Medicine disc).

According to the search strategy of this study, a total of 954 related studies were obtained in the initial examination, and 30 RCTs were included in quantitative synthesis ultimately [13–42]. The PRISMA flow diagram of studying selection is shown in Figure 1.

**Figure 2:**
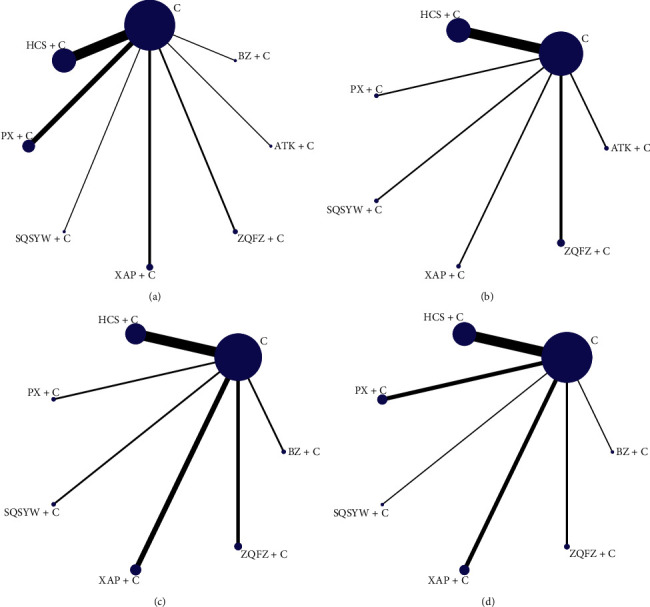
Network graph for 4 outcomes. (a) Objective response rate. (b) Performance status. (c) Leucopenia. (d) Gastrointestinal reaction.

There were 2602 patients in 30 studies, involving seven kinds of oral Chinese patent medicines, namely, Antike capsule (ATK), Bazhen granule (BZ), Shenqi Shiyiwei granule (SQSYW), Cinobufacin capsule (HCS), Pingxiao capsule (PX), Xiao'aiping tablet (XAP), and Zhenqi Fuzheng granule (ZQFZ). The number of RCTs related to these medicines was 1, 1, 1, 12, 6, 5, and 4, respectively. The experimental group contained 1342 cases, and the control group 1260 cases. Male patients accounted for 59.6%, and female patients accounted for 40.4%. All the studies reported the tumour-node-metastasis (TNM) stages and ages. There were 26 (86.7%), 13 (43.3%), 14 (46.7%), and 22 (73.3%) studies reported the ORR, KPS, leucopenia, and gastrointestinal reaction, respectively. Details of study characteristics are shown in Table 1. The network graph of 4 outcomes is presented in Figure 2. Since no closed loop is formed in each network graph, inconsistencies are not tested.

### 3.2. Risk of Bias within Studies

**Figure 3:**
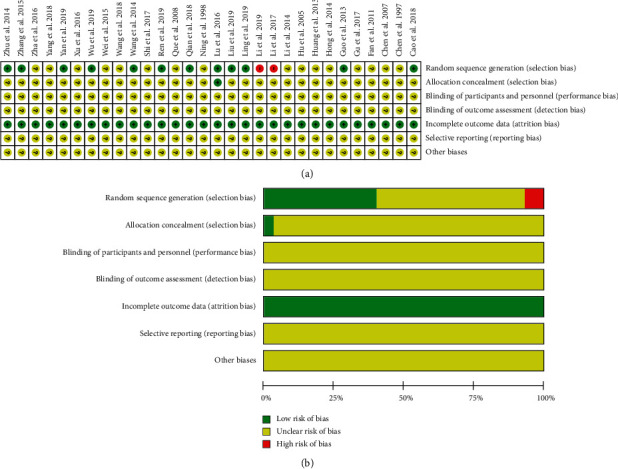
Risk of bias assessment.

In terms of random sequence generation, 12 of 30 studies used reasonable methods to generate the random sequence, including random number table, coin toss, and computer-generated random numbers, which were evaluated as “low risk of bias.” However, two studies were grouped by treatments, which were “high risk of bias,” and the rest mentioned only random sequences, which were “unclear risk of bias.” In terms of allocation concealment, only 1 study mentioned the use of orderly sealed envelope for allocation concealment, which was “low risk of bias,” and the other studies did not report the information of allocation concealment. None of the studies mentioned the information on blinding. All of the studies reported complete outcome data and belonged to a “low risk of bias.” Whether there are selective reporting results and other biases in all studies, which could not be clearly judged according to the literature information, belongs to “unclear risk of bias.” The results of bias risk assessment for all included studies are presented in Figure 3.

### 3.3. Results of the Meta-Analysis

The following C represents chemotherapy to simplify the expression of the results.

#### 3.3.1. Objective Response Rate

**Figure 4:**
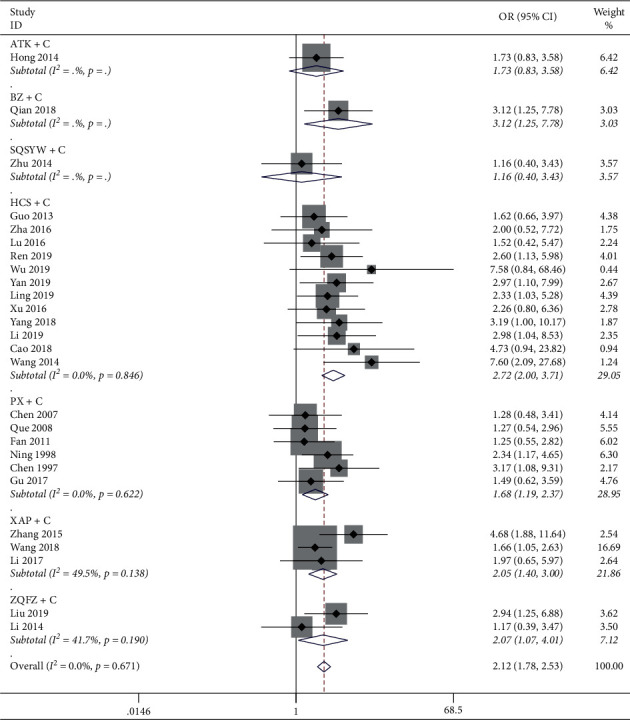
Meta-analysis results of the objective response rate.

The results of the heterogeneity test showed that the heterogeneity between the 26 studies was small (*P*=0.671, *I*^2^ = 0.0%), and *I*^2^ in each subgroup was all less than 50%. The results of the meta-analysis of the fixed-effect model showed that the effective rate of BZ + C, HCS + C, PX + C, XAP + C, and ZQFZ + C in the treatment of gastric cancer was significantly higher than that of chemotherapy alone (*P* < 0.05), but there was no significant difference between ATC + C, SQSYW + C, and chemotherapy alone, as shown in Figure 4.

#### 3.3.2. Performance Status

**Figure 5:**
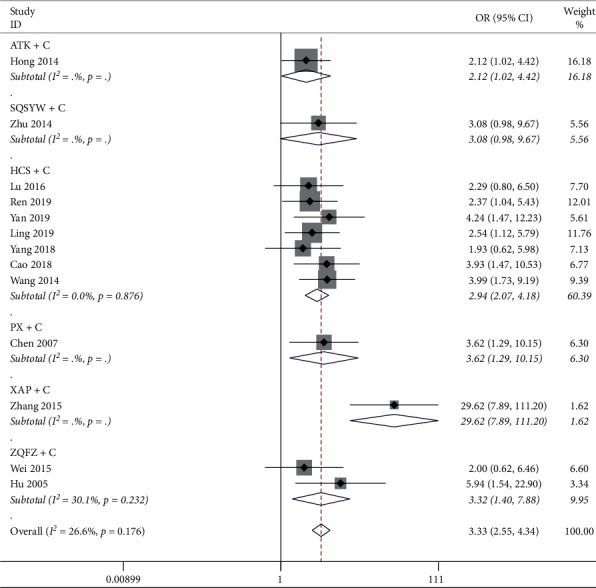
Meta-analysis results of performance status.

The results of the heterogeneity test showed that the heterogeneity between 13 studies was relatively small (*P*=0.176, *I*^2^ = 26.6%), and *I*^2^ in each subgroup was also less than 50%. The results of the meta-analysis of the fixed-effect model showed that ATC + C, HCS + C, PX + C, XAP + C, and ZQFZ + C could significantly improve the quality of life of patients with gastric cancer compared with chemotherapy alone (*P* < 0.05), while SQSYW + C had no significant difference compared with chemotherapy alone, as shown in Figure 5.

#### 3.3.3. ADRs

**Figure 6:**
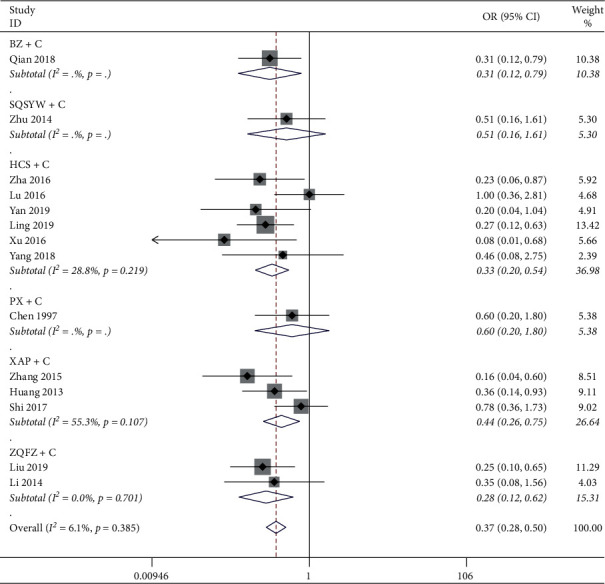
Meta-analysis results of leucopenia.

*(1) Leucopenia.* The results of the heterogeneity test showed that the heterogeneity among 14 studies was small (*P*=0.385, *I*^2^ = 6.1%), but *I*^2^ > 50% in the XAP + C subgroup, so the random effect model was used for analysis. The results of the meta-analysis showed that BZ + C, HCS + C, XAP + C, and ZQFZ + C could significantly reduce the incidence of leucopenia compared with chemotherapy alone (*P* < 0.05), while SQSYW + C and PX + C had no significant difference compared with chemotherapy alone, as shown in Figure 6.

**Figure 7:**
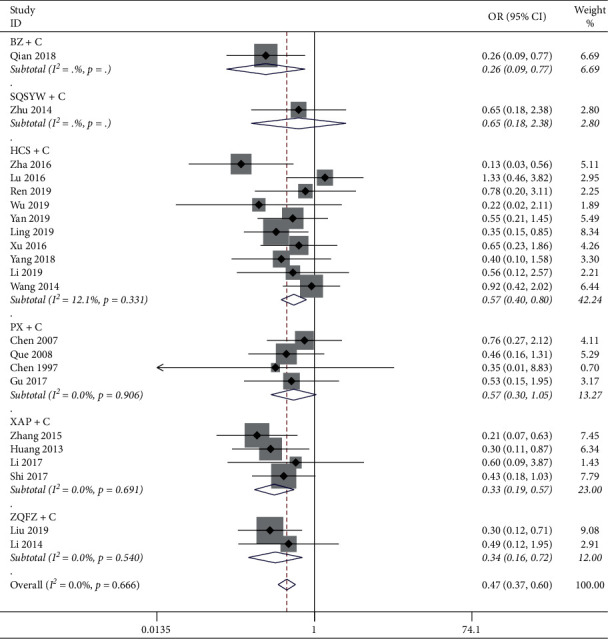
Meta-analysis results of gastrointestinal reaction.

*(2) Gastrointestinal Reaction.* The results of the heterogeneity test showed that the heterogeneity of 22 studies was little (*P*=0.666, *I*^2^ = 0%), and the heterogeneity within each subgroup was also small. The results of the meta-analysis of the fixed-effect model showed that BZ + C, HCS + C, XAP + C, and ZQFZ + C could significantly reduce the incidence of gastrointestinal reactions compared with chemotherapy alone (*P* < 0.05), but SQSYW + C and PX + C had no significant difference compared with chemotherapy alone, as shown in Figure 7.

### 3.4. Results of the Network Meta-Analysis

#### 3.4.1. Objective Response Rate

A total of 26 RCTs involving seven oral Chinese patent medicines reported ORR. The results of network meta-analysis showed that: compared with chemotherapy alone, BZ + C (OR = 0.31, 95% CI 0.11 to 0.87), HCS + C (OR = 2.78, 95% CI 1.97 to 3.95), PX + C (OR = 1.69, 95% CI 1.13 to 2.56), and XAP + C (OR = 2.20, 95% CI 1.37 to 3.77) can significantly improve the objective response rate and clinical efficacy. However, there were no significant differences between different oral Chinese patent medicines. The effect value of each intervention is shown in Table 2.

**Figure 8:**
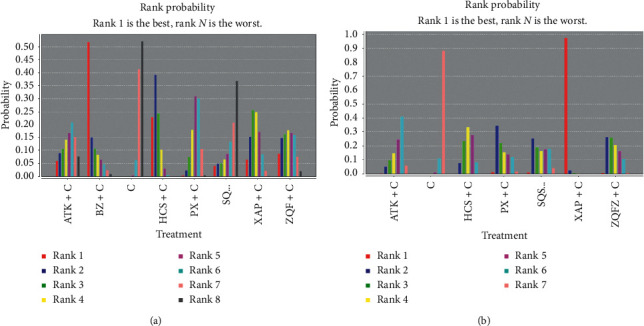
The ranking probability of each intervention. *Note.* (a) Objective response rate. (b) Performance status.

The rank probability of interventions is represented in Figure 8. For each intervention, the total rank probability was 1. In terms of ORR, rank 1 was the best intervention, while rank *N* was worst. The rank of oral Chinese patent medicines was BZ (1 RCT) > HCS (12 RCTs) > XAP (3 RCTs) > ZQFZ (2 RCTs) > PX (6 RCTs) > ATK (1 RCT) > SQSYW (1 RCT).

#### 3.4.2. Performance Status

A total of 13 RCTs involving six oral Chinese patent medicines reported the improvement rate of the KPS score. There were significant differences between HCS + C (OR = 0.33, 95% CI 0.21 to 0.51), PX + C (OR = 0.27, 95% CI 0.07 to 0.91), XAP + C (OR = 0.03, 95% CI 0.00 to 0.12), ZQFZ + C (OR = 0.28, 95% CI 0.10 to 0.77) and chemotherapy alone. Besides, the performance status of the patients with gastric cancer improved by the combination of XAP and chemotherapy was significantly better than the other five oral Chinese patent medicines. There is no statistically significant difference between the other interventions, as shown in Table 2.

The rank probability of each intervention is displayed in Figure 8. As same as ORR, rank 1 represented the best effect here. The rank of oral Chinese patent medicines was: XAP (1 RCT) > PX (1 RCT) > SQSYW (1 RCT) > HCS (7 RCTs) > ZQFZ (2 RCTs) > ATK (1 RCT).

#### 3.4.3. ADRs

*(1) Leucopenia.* A total of 14 RCTs involving six oral Chinese patent medicines reported the incidence of leucopenia. The results showed that only the combination of HCS and chemotherapy (OR = 0.30, 95% CI 0.13 to 0.65) could significantly reduce the incidence of leucopenia compared with chemotherapy alone. There is no significant difference between the other interventions, as shown in Table 3.

**Figure 9:**
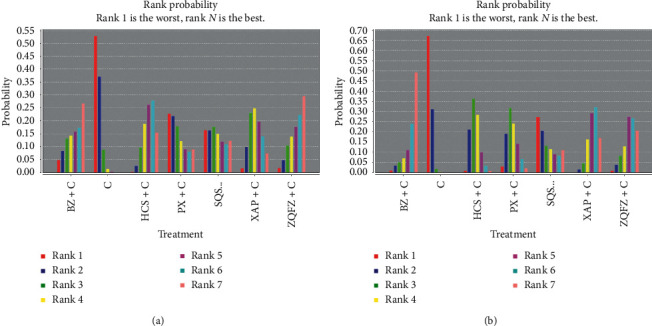
The rank probability of each intervention. (a) Leucopenia. (b) Gastrointestinal reaction.

The rank probability of each intervention was presented in Figure 9. In terms of leucopenia, a larger portion of rank 6 represented better effects, while rank 1 was the worst. The rank of oral Chinese patent medicines was ZQFZ (2 RCTs) > BZ (1 RCT) > HXS (6 RCTs) > SQSYW (1 RCT) > PX (1 RCT) > XAP (3 RCTs).

*(2) Gastrointestinal Reaction.* A total of 22 RCTs involving six oral Chinese patent medicines provided the data of gastrointestinal response. The results showed that BZ + C (OR = 0.26, 95% CI 0.07 to 0.83), XAP + C (OR = 2.95, 95% CI 1.65 to 5.88), and ZQFZ + C (OR = 2.92, 95% CI 1.24 to 7.06) can significantly reduce the incidence of gastrointestinal response compared with chemotherapy alone. There is no significant difference between the other interventions, as shown in Table 3.

The rank probability of each intervention was displayed in Figure 9. Like leucopenia, rank 6 was the best. The rank of oral Chinese patent medicines was BZ (1 RCT) > ZQFZ (1 RCT) > XAP (4 RCTs) > SQSYW (1 RCT) > PX (4 RCTs) > HCS (10 RCTs).

### 3.5. Publication Bias

**Figure 10:**
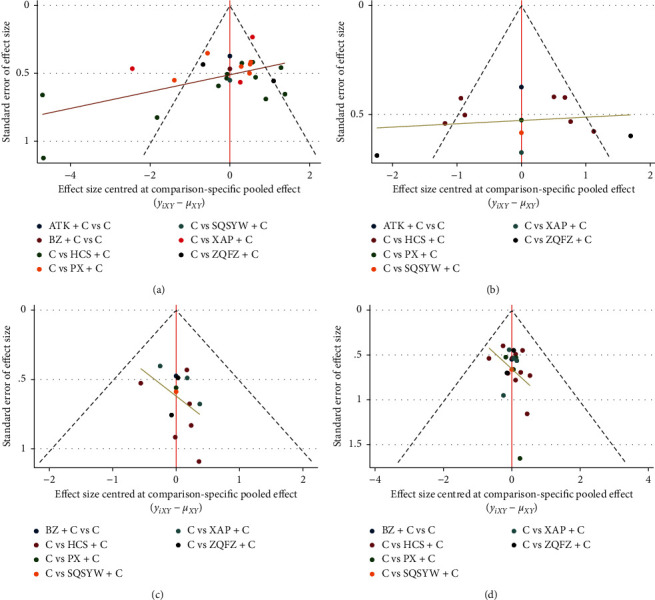
Funnel plot for 4 outcomes. (a) Objective response rate. (b) Performance status. (c) Leucopenia. (d) Gastrointestinal reaction.

The publication bias of the RCTs was measured with a comparison-adjusted funnel plot. Funnel plots of most outcomes were not quite symmetric, indicating potential publication bias in the network (Figure 10).

## 4. Discussion

As a common malignant tumour, gastric cancer has a high incidence and recurrence rate, and its prevention and control have become an urgent public health issue. Traditional Chinese medicine has been extensively applied in China and has shown certain advantages in the treatment of gastric cancer. At present, relevant studies in China have used network meta-analysis to evaluate the efficacy and safety of Chinese herbal injections combined with chemotherapy in the treatment of gastric cancer, respectively, from the aspects of short-term efficacy, quality of life, the incidence of adverse reactions, and so on, to help clinicians choose the best scheme in different interventions [43–46]. However, the network meta-analysis of traditional Chinese medicine combined with chemotherapy in the treatment of gastric cancer is mostly aimed at Chinese herbal injections, and the direction of oral Chinese patent medicine combined with chemotherapy has not been involved. The medicines listed in this study are commonly used oral Chinese patent medicines for gastric cancer recommended in the *Clinical Practice Guidelines of Chinese Medicine in Oncology*, and these medicines are included in the National Basic Medical Insurance Drugs List of China. According to the eligibility criteria, this NMA identified 30 RCTs involving seven oral Chinese patent medicines, namely, HCS, PX, XAP, ZQFZ, BZ, ATK, and SQSYW. In this study, the efficacy and safety of 7 kinds of oral Chinese patent medicine combined with chemotherapy in the treatment of gastric cancer were compared. The results of the network meta-analysis showed the following. (1) In terms of improving the objective response rate, compared with chemotherapy alone, BZ + C (OR = 0.31, 95% CI 0.11 to 0.87), HCS + C (OR = 2.78, 95% CI 1.97 to 3.95), PX + C (OR = 1.69, 95% CI 1.13 to 2.56), and XAP + C (OR = 2.20, 95% CI 1.37 to 3.77) can significantly improve the objective response rate and clinical efficacy. However, there was no significant difference between different oral Chinese patent medicines. The results of probability ranking show that the top three are BZ + C, HCS + C, and XAP + C, respectively. (2) In terms of improving performance status, there were significant differences between HCS + C (OR = 0.33, 95% CI 0.21 to 0.51), PX + C (OR = 0.27, 95% CI 0.07 to 0.91), XAP + C (OR = 0.03, 95% CI 0.00 to 0.12), ZQFZ + C (OR = 0.28, 95% CI 0.10 to 0.77), and chemotherapy alone. The pair-pair comparison of oral Chinese patent medicine showed that XAP + C improved the quality of life of gastric cancer patients significantly better than ATK + C, HCS + C, PX + C, SQSYW + C, and ZQFZ + C. In contrast, other pair-pair comparison showed no statistical significance. The probability ranking results show that the top three are XAP + C, PX + C, and SQSYW + C, respectively. (3) In terms of reducing toxic and side effects, HCS + C (OR = 0.30, 95% CI 0.13 to 0.65) can significantly reduce the incidence of leucopenia, and there was no significant difference between the other two interventions. The probability ranking results show that the top three are ZQFZ + C, BZ + C, and HCS + C, respectively; BZ + C (OR = 0.26, 95% CI 0.07 to 0.83), XAP + C (OR = 2.95, 95% CI 1.65 to 5.88), and ZQFZ + C (OR = 2.92, 95% CI 1.24 to 7.06) can significantly reduce the incidence of gastrointestinal reactions. There was no significant difference between the other two interventions. The probability ranking results show that the top three are BZ + C, ZQFZ + C, and XAP + C, respectively.

The results of this study suggest that Bazhen granule combined with chemotherapy in the treatment of gastric cancer may be the best way to improve the clinical efficacy and reduce the adverse reactions of chemotherapy compared with the other six kinds of Chinese patent medicine. However, Bazhen granule is limited by the number and sample size of the included study, and its statistical test efficacy is low. Therefore, based on the current evidence, we suggest that clinicians should make the best choice according to different therapeutic purposes when using oral Chinese patent medicine combined with chemotherapy to treat gastric cancer. When the purpose is to improve the chemotherapy efficiency of gastric cancer patients, it is recommended to choose Cinobufacin capsule first; when the purpose is to improve the performance status of patients, it is recommended to select Xiao'aiping tablet first; when the purpose is to reduce the side effects of chemotherapy, it is recommended to choose Bazhen granule or Zhenqi Fuzheng granule.

In recent years, a lot of achievements have been recorded in clinical and experimental research on the treatment of gastric cancer with traditional Chinese medicine. Cinobufacin is a Traditional Chinese medicine *Bufo gargarizans* or *Bufo melanostictus* Schneider skin aqueous preparation, and its main active ingredient is bufogenin. It has the pharmacological effects of antitumour and immune promotion [47]. Studies on its antitumour mechanism have shown that Cinobufacin can inhibit tumour cell proliferation, induce tumour cell apoptosis, inhibit tumour angiogenesis, enhance immune function, and so on [48]. Its related preparations are widely used in the treatment of gastric cancer and other malignant tumours and show good efficacy. Xiao'aiping tablet is a kind of oral Chinese patent medicine commonly used by gastric cancer patients. Its core component is Marsdenia Tenacissima Caulis, which has the effect of preventing tumour cells from mitosis and promoting tumour cells apoptosis [49]. Modern pharmacological research also showed that Xiao'aiping tablet could reduce the content of transforming growth factor-*α* (TGF-*α*) and vascular endothelial growth factor (VEGF) in patients with gastric cancer, to inhibit the invasion, metastasis, and angiogenesis of gastric cancer cells [50]. Zhenqi Fuzheng granule is an oral preparation composed of Astragali Radix and Ligustri Lucidi Fructus. It has the effects of improving human immunity, supporting the normal and tonifying the deficiency, tonifying qi and nourishing yin, and improving leucopenia caused by radiotherapy and chemotherapy in cancer patients. Bazhen granule is a Chinese patent medicine composed of Paeoniae Radix Alba, Atractylodis Macrocephalae Rhizoma, Atractylodes Macrocephala, Chuanxiong Rhizoma, Angelicae Sinensis Radix, Poria, Glycyrrhizae Radix Et Rhizoma, and Rehmanniae Radix Preaparata, which has the effect of tonifying qi and blood. The research shows that Bazhen granules can regulate the imbalance of many trace elements in the body and improve the immune function of patients with malignant tumours, especially those with deficiency of qi and blood [51]. In general, different types of oral Chinese patent medicines have diverse effects and functions on gastric cancer patients. In the clinical application of such drugs, we should combine the experience of doctors, the situation of patients, and a high level of evidence-based medicine research to choose.

This study takes the initiative to compare the efficacy and safety of a variety of oral Chinese patent medicines in the treatment of gastric cancer. However, the limitations of this study should not be ignored: ① the included studies are all Chinese literature, and there may be language bias; ② the quality of the included studies is general, most of the random methods are not explicitly explained, and most of the studies do not provide information about the distribution concealment and blind method, which may affect the reliability of the results; ③ funnel plot results suggest that there is a higher possibility of publication bias, which may affect the authenticity of the results; ④ there is a lack of direct comparative study between different oral Chinese patent medicines, and the confidence interval is wide, which may affect the statistical efficacy; ⑤ limited by the specific chemotherapy regimen, it failed to evaluate the effectiveness of the same oral Chinese patent medicine combined with different chemotherapy regimens; ⑥ limited by the included study, the selected outcome is the short-term efficacy index, and it failed to evaluate the long-term efficacy for gastric cancer; ⑦ the studies included in the analysis are all conducted in Chinese population. It is not clear whether our conclusions apply to other populations. Therefore, in the future, multicentre, large sample, and high-quality research can be organized to further clarify the effectiveness and safety of oral Chinese patent medicine in the treatment of gastric cancer and define its clinical feasibility, to achieve the initial purpose of guiding clinical practice.

## 5. Conclusions

In conclusion, oral Chinese patent medicine can play a useful role in enhancing the efficacy and reducing the toxicity of chemotherapy in gastric cancer patients. Among the seven drugs, Cinobufacin capsule and Xiao'aiping tablet are the best choices in improving the clinical efficacy, and Bazhen granule and Zhenqi Fuzheng granule are the best choices in reducing the adverse reactions of chemotherapy. Bazhen granule showed a good effect in this network meta-analysis. In the future, we should pay more attention to the effect of Bazhen granule combined with chemotherapy in the treatment of gastric cancer. Given the limitations of this study, the application of the conclusions of this study should be carefully selected.

## Figures and Tables

**Table 1:** The basic characteristics of the included studies.

Study ID	TNM stages	Sample size (E/C)	Sex, M/F	Age (E/C)	Intervention		Course (d × c)	Outcomes
					E	C		
Hong et al. [13]	III∼IV	60/60	79/41	58.0/56.5	ATK 0.44 g, tid + SOX	SOX	≥21d × 2	①②
Qian and Zuo [14]	III∼IV	40/40	59/21	62.5(19∼74)/62(38∼77)	BZ 3.5 g, bid + 5-Fu + THP + L-OHP	5-Fu + THP + L-OHP	21 d × 3	①③④
Zhu et al. [15]	III∼IV	27/27	36/18	61(19∼73)/58(24∼75)	SQSYW 2g, tid + EOX	EOX	≥21 d × 2	①②③④
Guo et al. [16]	III∼IV	42/38	40/40	66.4 ± 4.2/64.8 ± 3.7	HCS 0.9 g,qid + FOLFOX6	FOLFOX6	21 d × 6	①
Zha and Hang [17]	III∼IV	20/20	24/16	50∼72	HCS 0.5 g,tid + oxaliplatin 130 mg/m^2^ + Tegafur 600 mg/m^2^ + CF 200 mg/m^2^	Oxaliplatin 130 mg/m^2^ + Tegafur 600 mg/m^2^ + CF 200 mg/m^2^	21 d × 6	①③④
Lu et al. [18]	III∼IV	30/30	23/37	73.7 ± 5.1/74.8 ± 6.2	HCS 0.5 g, tid + capecitabine 1250 mg/m^2^	Capecitabine 1250 mg/m^2^	≥21 d × 2	①②③④
Ren [19]	III∼IV	47/47	54/40	51.24 ± 3.98/50.15 ± 3.87	HCS 0.5 g, tid + SOX	SOX	21 d × 2	①②④
Wu et al. [20]	IV	25/25	27/23	59.14 ± 4.37/58.57 ± 4.23	HCS 0.5 g, tid + XELOX	XELOX	21 d × 2	①④
Yan et al. [21]	IIIB∼IV	35/35	38/32	49.5 ± 6.4/48.76 ± 6.5	HCS 0.9 g, tid + oxaliplatin 85 mg/m^2^ + capecitabine 1000 mg/m^2^	Oxaliplatin 85 mg/m^2^ + capecitabine 1000 mg/m^2^	21 d × 6	①②③④
Ling [22]	III∼IV	48/48	61/35	54.27 ± 7.92/55.03 ± 7.51	HCS 0.9 g, tid + FOLFOX4	FOLFOX4	14 d × 3	①②③④
Xu and Liu [23]	III∼IV	30/30	32/28	45. 8(36∼70)/49. 9(37∼70)	HCS 0.9 g, tid + L-OHP 130 mg/m^2^ + 5-FU 300 mg/m^2^ + CF 200 mg/m^2^	L-OHP 130 mg/m^2^ + 5-FU 300 mg/m^2^ + CF 200 mg/m^2^	≥21 d × 2	①③④
Yang and Zhang [24]	IV	25/25	35/15	54(31∼75)/50(37∼70)	HCS 0.5 g,tid + EOF	EOF	≥21 d × 2	①②③④
Li et al. [25]	III∼IV	30/30	38/22	61.5 ± 9.0/60.8 ± 8.8	HCS 0.9 g, tid + SOX	SOX	21 d × 2	①④
Cao [26]	NR	41/41	44/38	54.8 ± 5.4/56.3 ± 4.6	HCS 0.9 g, tid + S-1 60 mg/m^2^	S-1 60 mg/m^2^	28 d × 2	①②
Wang and et al. [27]	NR	58/58	59/57	58. 4/58. 8	HCS 0.5 g, tid + S-1 80 mg/m^2^	S-1 80 mg/m^2^	21 d × 2	①②④
Chen et al. [28]	I∼IV	33/33	36/30	48.8	PX 1.15∼1.84 g, tid + ELF	ELF	21 d × 2	①②④
Que and Wang [29]	NR	44/43	49/38	52(35∼69)	PX 1.38 g, tid + DDP + 5-Fu	DDP + 5-Fu	21 d × 2	①④
Fan et al. [30]	III∼IV	47/46	55/38	52(32∼69)	PX 1.25 g, bid + DDP + 5-Fu	DDP + 5-Fu	21 d × 1	①
Ning and Hao [31]	II∼IV	121/49	126/44	54.3/56.1	PX 1.68 g, tid + mFAM	mFAM	≥21 d × 3	①
Chen et al. [32]	III∼IV	30/28	43/15	51(32∼69)/50(29∼68)	PX 1.38 g,tid + ECF	ECF	21 d × 2	①③④
Gu [33]	IV	50/50	61/39	45.81 ± 8.79/45.17 ± 8.92	PX 1.15 g, tid + DCF	DCF	21 d × 4	①④
Zhang et al. [34]	III∼IV	46/46	61/31	54.3 ± 6.8/52.6 ± 6.3	XAP 3 g, bid + PF	PF	28 d × 2	①②③④
Huang and Guo [35]	NR	36/36	41/31	61.42 ± 11.20	XAP 2.4 g, tid + FOLFOX4/XELOX/EOF	FOLFOX4/XELOX/EOF	NR	③④
Wang [36]	NR	150/150	166/134	62.34 ± 8.37/63.16 ± 8.84	XAP 1.8∼2.4 g, tid + SOX	SOX	21 d × 4	①
Li [37]	IV	32/30	37/25	62.2 ± 3.4/63.6 ± 3.2	XAP + chemotherapy	Chemotherapy	NR	①④
Shi [38]	NR	53/53	56/50	56.28 ± 4.82	XAP 2.04∼2.55 g, tid + EOF/OLF	EOF/OLF	NR	③④
Liu [39]	III	46/46	57/35	57.2 ± 4.1/56.1 ± 3.5	ZQFZ 15 g, bid + FOLFOX4	FOLFOX4	≥14 d × 2	①③④
Wei [40]	NR	30/30	34/26	43∼72/46∼75	ZQFZ 5g, bid + 5-Fu + L-OHP + CF + MMC	5-Fu + L-OHP + CF + MMC	28d × 1	②
Hu et al. [41]	II∼IV	40/40	46/34	54.3 ± 10.3/52.5 ± 11.1	ZQFZ 5g, tid + MLF	MLF	28 d × 1	②
Li and Zhang [42]	IV	26/26	34/18	65∼73	ZQFZ 5g, bid + S-1	S-1	42 d × 2	①③④

E, experimental group; C, control group; M, male; F, female; NR, no reported; d, day; c, cycle; ATK, Antike capsule; BZ, Bazhen granule; SQSYW, Shenqi Shiyiwei granule; HCS, Cinobufacin capsule; PX, Pingxiao capsule; XAP, Xiao'aiping tablet; ZQFZ, Zhenqi Fuzheng granule; ①, objective response rate; ②, performance status; ③, leucopenia; ④, gastrointestinal reaction.

**Table 2:** Network meta-analysis results of objective response rate (upper right quarter) and performance status (lower left quarter).

ATK + C	1.87 (0.47, 7.30)	0.58 (0.24, 1.39)	1.60 (0.62, 4.21)	0.99 (0.36, 2.55)	0.69 (0.14, 3.18)	1.27 (0.47, 3.58)	1.19 (0.37, 3.79)
–	**BZ** + **C**	*0.31 (0.11, 0.87)*	0.86 (0.29, 2.60)	0.53 (0.17, 1.59)	0.38 (0.07, 1.77)	0.69 (0.22, 2.20)	0.63 (0.16, 2.27)
2.13 (0.76, 6.30)	—	**C**	*2.78 (1.97, 3.95)*	*1.69 (1.13, 2.56)*	1.20 (0.33, 4.10)	*2.20 (1.37, 3.77)*	2.04 (0.97, 4.33)
0.71 (0.23, 2.33)	—	*0.33 (0.21, 0.51)*	**HCS** + **C**	0.61 (0.35, 1.04)	0.43 (0.12, 1.53)	0.79 (0.44, 1.48)	0.73 (0.33, 1.67)
0.59 (0.11, 2.85)	—	*0.27 (0.07, 0.91)*	0.81 (0.19, 3.01)	**PX** + **C**	0.70 (0.19, 2.58)	1.30 (0.70, 2.57)	1.21 (0.50, 2.85)
0.65 (0.12, 3.91)	—	0.31 (0.08, 1.16)	0.91 (0.22, 3.71)	1.14 (0.18, 7.65)	**SQSYW** + **C**	1.83 (0.49, 7.51)	1.71 (0.38, 7.20)
*0.06 (0.01, 0.39)*	—	*0.03 (0.00, 0.12)*	*0.09 (0.01, 0.40)*	*0.11 (0.01, 0.80)*	*0.09 (0.01, 0.73)*	**XAP** + **C**	0.92 (0.37, 2.25)
0.62 (0.14, 2.59)	—	*0.28 (0.10, 0.77)*	0.84 (0.28, 2.61)	1.09 (0.22, 5.76)	0.97 (0.16, 5.09)	*10.12 (1.59, 85.37)*	**ZQFZ** + **C**

The values in italics indicate there is a significant difference between the two groups.

**Table 3:** Results of the network meta-analysis for leucopenia (upper right quarter) and gastrointestinal reaction (lower left quarter).

BZ + C	3.24 (0.57, 19.51)	0.96 (0.14, 6.41)	1.93 (0.15, 23.60)	1.53 (0.12, 20.54)	1.28 (0.16, 9.39)	0.88 (0.10, 8.09)

*0.26 (0.07, 0.83)*	**C**	*0.30 (0.13, 0.65)*	0.60 (0.09, 3.59)	0.48 (0.07, 3.12)	0.40 (0.13, 1.05)	0.27 (0.07, 1.04)
0.46 (0.12, 1.68)	1.79 (1.17, 2.84)	**HCS + C**	2.01 (0.26, 15.23)	1.62 (0.23, 13.25)	1.35 (0.36, 4.86)	0.91 (0.20, 4.58)
0.47 (0.11, 1.84)	1.85 (0.94, 3.90)	1.03 (0.44, 2.41)	**PX + C**	0.81 (0.06, 11.46)	0.67 (0.08, 5.54)	0.46 (0.05, 4.65)
0.40 (0.05, 2.67)	1.52 (0.36, 6.58)	0.85 (0.18, 3.81)	0.83 (0.17, 4.26)	**SQSYW + C**	0.83 (0.09, 6.08)	0.56 (0.06, 5.76)
0.73 (0.18, 3.04)	*2.95 (1.65, 5.88)*	1.65 (0.78, 3.58)	1.62 (0.62, 4.28)	1.99 (0.39, 9.86)	**XAP + C**	0.67 (0.14, 4.16)
0.74 (0.15, 3.08)	*2.92 (1.24, 7.06)*	1.61 (0.59, 4.34)	1.55 (0.49, 4.87)	1.88 (0.33, 11.05)	1.01 (0.32, 2.87)	**ZQFZ + C**

## Data Availability

The data used to support the findings of this study are included within the supplementary information files.

## Abbreviations

95% CI: 95% confidence interval
OR: Odds ratio
ADRs: Adverse drug reactions
NMA: Network meta-analysis
RCTs: Randomized controlled trials
IF: Inconsistency factor
CR: Complete response
PR: Partial response
ORR: Objective response rate
KPS: Karnofsky
ECOG: Eastern Cooperative Oncology Group
C: Chemotherapy
ATK: Antike capsule
BZ: Bazhen granule
SQSYW: Shenqi Shiyiwei granule
HCS: Cinobufacin capsule
PX: Pingxiao capsule
XAP: Xiao'aiping tablet
ZQFZ: Zhenqi Fuzheng granule.

## Supplementary Materials

Supplementary Materials Table S1: PRISMA NMA Checklist. Table S2: details of the search strategy for PubMed.
