# Supplementary material for: Effectiveness and Safety of Oral Chinese Patent Medicines Combined with Chemotherapy for Gastric Cancer: A Bayesian Network Meta-Analysis
Source: Evid Based Complement Alternat Med. 2020 Aug 26;2020:8016531. doi: 10.1155/2020/8016531 (PMC7471790; doi:10.1155/2020/8016531)
Supplement: Supplementary Materials — Table S1: PRISMA NMA Checklist. Table S2: details of the search strategy for PubMed. [file 8016531.f1.zip › Table S2:Details of the search strategy for PubMed.docx]

**Table S2: Details of the search strategy for PubMed**

| **No. Search item** |
| --- |
| #1 Stomach Neoplasms[Mesh] |
| #2 Stomach Neoplasms OR Neoplasm, Stomach OR Stomach Neoplasm OR Neoplasms, Stomach OR Gastric Neoplasms OR Gastric Neoplasm OR Neoplasm, Gastric OR Neoplasms, Gastric OR Cancer of Stomach OR Stomach Cancers OR Gastric Cancer OR Cancer, Gastric OR Cancers, Gastric OR Gastric Cancers OR Stomach Cancer OR Cancer, Stomach OR Cancers, Stomach OR Cancer of the Stomach OR Gastric Cancer, Familial Diffuse |
| #3 #1 OR #2 |
| #4 bailing OR jianpiyishen OR zhenqifuzheng OR bazhen OR buzhongyiqi OR shiquandabu OR xiaoaiping OR huachansu OR cinobufacin OR antike OR pingxiao OR anduolin OR yangyinshengxue OR shiyiweishenqi OR shenqishiyiwei OR kanglaite |
| #5 Randomized Controlled Trial [Publication Type] |
| #6 Controlled Clinical Trial [Publication Type] |
| #7 randomized OR randomly OR placebo |
| #8 #5 OR #6 OR #7 |
| #9 #3 AND #4 AND #8 |
